# The Madrid Statement on Poly- and Perfluoroalkyl Substances (PFASs)

**DOI:** 10.1289/ehp.1509934

**Published:** 2015-05-01

**Authors:** Arlene Blum, Simona A. Balan, Martin Scheringer, Xenia Trier, Gretta Goldenman, Ian T. Cousins, Miriam Diamond, Tony Fletcher, Christopher Higgins, Avery E. Lindeman, Graham Peaslee, Pim de Voogt, Zhanyun Wang, Roland Weber

**Affiliations:** 1 Department of Chemistry, University of California at Berkeley, Berkeley, California, USA; 2 Green Science Policy Institute, Berkeley, California, USA; 3 Leuphana University, Lüneburg, Germany; 4 Safety and Environmental Technology Group, Institute for Chemical and Bioengineering, ETH Zürich, Zürich, Switzerland; 5 Division of Food Chemistry, National Food Institute, Technical University of Denmark, Kongens Lyngby, Denmark; 6 European Centre on Sustainable Policies for Human and Environmental Rights, Brussels, Belgium; 7 Department of Applied Environmental Science, Stockholm University, Stockholm, Sweden; 8 Department of Earth Sciences, University of Toronto, Toronto, Ontario, Canada; 9 Department of Social and Environmental Health Research, London School of Hygiene & Tropical Medicine, London, United Kingdom; 10 Department of Civil and Environmental Engineering, Colorado School of Mines, Golden, Colorado, USA; 11 Chemistry Department, Hope College, Holland, Michigan, USA; 12 Institute for Biodiversity and Ecosystem Dynamics, University of Amsterdam, Amsterdam, the Netherlands; 13 POPs Environmental Consulting, Schwäbisch Gmünd, Germany

As scientists and other professionals from a variety of disciplines, we are concerned about the production and release into the environment of an increasing number of poly- and perfluoroalkyl substances (PFASs) for the following reasons:

PFASs are man-made and found everywhere. PFASs are highly persistent, as they contain perfluorinated chains that only degrade very slowly, if at all, under environmental conditions. It is documented that some polyfluorinated chemicals break down to form perfluorinated ones (D’Eon and Mabury 2007).
PFASs are found in the indoor and outdoor environments, wildlife, and human tissue and bodily fluids all over the globe. They are emitted via industrial processes and military and firefighting operations (Darwin 2011; Fire Fighting Foam Coalition 2014), and they migrate out of consumer products into air (Shoeib et al. 2011), household dust (Björklund et al. 2009), food (Begley et al. 2008; Tittlemier et al. 2007; Trier et al. 2011), soil (Sepulvado et al. 2011; Strynar et al. 2012), ground and surface water, and make their way into drinking water (Eschauzier et al. 2012; Rahman et al. 2014).
In animal studies, some long-chain PFASs have been found to cause liver toxicity, disruption of lipid metabolism and the immune and endocrine systems, adverse neurobehavioral effects, neonatal toxicity and death, and tumors in multiple organ systems (Lau et al. 2007; Post et al. 2012). In the growing body of epidemiological evidence, some of these effects are supported by significant or suggestive associations between specific long-chain PFASs and adverse outcomes, including associations with testicular and kidney cancers (Barry et al. 2013; Benbrahim-Tallaa et al. 2014), liver malfunction (Gallo et al. 2012), hypothyroidism (Lopez-Espinosa et al. 2012), high cholesterol (Fitz-Simon et al. 2013; Nelson et al. 2009), ulcerative colitis (Steenland et al. 2013), lower birth weight and size (Fei et al. 2007), obesity (Halldorsson et al. 2012), decreased immune response to vaccines (Grandjean et al. 2012), and reduced hormone levels and delayed puberty (Lopez-Espinosa et al. 2011).
Due to their high persistence, global distribution, bioaccumulation potential, and toxicity, some PFASs have been listed under the Stockholm Convention (United Nations Environment Programme 2009) as persistent organic pollutants (POPs).
As documented in the Helsingør Statement (Scheringer et al. 2014),
Although some of the long-chain PFASs are being regulated or phased out, the most common replacements are short-chain PFASs with similar structures, or compounds with fluorinated segments joined by ether linkages.
While some shorter-chain fluorinated alternatives seem to be less bioaccumulative, they are still as environmentally persistent as long-chain substances or have persistent degradation products. Thus, a switch to short-chain and other fluorinated alternatives may not reduce the amounts of PFASs in the environment. In addition, because some of the shorter-chain PFASs are less effective, larger quantities may be needed to provide the same performance.
While many fluorinated alternatives are being marketed, little information is publicly available on their chemical structures, properties, uses, and toxicological profiles.
Increasing use of fluorinated alternatives will lead to increasing levels of stable perfluorinated degradation products in the environment, and possibly also in biota and humans. This would increase the risks of adverse effects on human health and the environment.
Initial efforts to estimate overall emissions of PFASs into the environment have been limited due to uncertainties related to product formulations, quantities of production, production locations, efficiency of emission controls, and long-term trends in production history (Wang et al. 2014).
The technical capacity to destroy PFASs is currently insufficient in many parts of the world.

Global action through the Montreal Protocol (United Nations Environment Programme 2012) successfully reduced the use of the highly persistent ozone-depleting chlorofluorocarbons (CFCs), thus allowing for the recovery of the ozone layer. However, many of the organofluorine replacements for CFCs are still of concern due to their high global warming potential. It is essential to learn from such past efforts and take measures at the international level to reduce the use of PFASs in products and prevent their replacement with fluorinated alternatives in order to avoid long-term harm to human health and the environment.

For these reasons, we call on the international community to cooperate in limiting the production and use of PFASs and in developing safer nonfluorinated alternatives. We therefore urge scientists, governments, chemical and product manufacturers, purchasing organizations, retailers, and consumers to take the following actions:

## Scientists:

Assemble, in collaboration with industry and governments, a global inventory of all PFASs in use or in the environment, including precursors and degradation products, and their functionality, properties, and toxicology.
Develop analytical methods for the identification and quantification of additional families of PFASs, including fluorinated alternatives.
Continue monitoring for legacy PFASs in different matrices and for environmental reservoirs of PFASs.
Continue investigating the mechanisms of toxicity and exposure (e.g., sources, fate, transport, and bioaccumulation of PFASs), and improve methods for testing the safety of alternatives.
Bring research results to the attention of policy makers, industry, the media, and the public.

## Governments:

Enact legislation to require only essential uses of PFASs, and enforce labeling to indicate uses.
Require manufacturers of PFASs to
conduct more extensive toxicological testing,
make chemical structures public,
provide validated analytical methods for detection of PFASs, and
assume extended producer responsibility and implement safe disposal of products and stockpiles containing PFASs.
Work with industry to develop public registries of products containing PFASs.
Make public annual statistical data on production, imports, and exports of PFASs.
Whenever possible, avoid products containing, or manufactured using, PFASs in government procurement.
In collaboration with industry, ensure that an infrastructure is in place to safely transport, dispose of, and destroy PFASs and PFAS-containing products, and enforce these measures.

## Chemical manufacturers:

Make data on PFASs publicly available, including chemical structures, properties, and toxicology.
Provide scientists with standard samples of PFASs, including precursors and degradation products, to enable environmental monitoring of PFASs.
Work with scientists and governments to develop safe disposal methods for PFASs.
Provide the supply chain with documentation on PFAS content and safe disposal guidelines.
Develop nonfluorinated alternatives that are neither persistent nor toxic.

## Product manufacturers:

Stop using PFASs where they are not essential or when safer alternatives exist.
Develop inexpensive and sensitive PFAS quantification methods for compliance testing.
Label products containing PFASs, including chemical identity and safe disposal guidelines.
Invest in the development and use of nonfluorinated alternatives.

## Purchasing organizations, retailers, and individual consumers:

Whenever possible, avoid products containing, or manufactured using, PFASs. These include many products that are stain-resistant, waterproof, or nonstick.
Question the use of such fluorinated “performance” chemicals added to consumer products.

## Signatories

**Ovokeroye Abafe**, Researcher, School of Chemistry and Physics, University of Kwazulu-Natal, Durban, South Africa **Marlene Ågerstrand**, PhD, Researcher, Department of Applied Environmental Science, Stockholm University, Stockholm, Sweden **Lutz Ahrens**, PhD, Research Scientist, Department of Aquatic Sciences and Assessment, Swedish University of Agricultural Sciences, Uppsala, Sweden **Beatriz H. Aristizabal**, PhD, Professor, Department of Chemical Engineering, National University of Colombia, Manizales, Colombia **Abel Arkenbout**, PhD, Chairman, ToxicoWatch Foundation, Harlingen, the Netherlands **Misha Askren**, MD, Physician, Urgent Care, Kaiser Permanente, Los Angeles, California, USA **Jannicke Bakkejord**, Senior Engineer, National Institute of Nutrition and Seafood Research, Bergen, Norway **Georg Becher**, PhD, Professor Emeritus, Department of Exposure and Risk Assessment, Norwegian Institute of Public Health, Oslo, Norway **Thea Bechshoft**, PhD, Postdoctoral Fellow, University of Southern Denmark, Odense, Denmark **Peter Behnisch**, PhD, Director, BioDetection System, Amsterdam, the Netherlands **Susanne Bejerot**, MD, Assistant Professor, Department of Clinical Neuroscience, Karolinska Institute, Stockholm, Sweden **Stephen Bent**, MD, Associate Professor of Medicine, Epidemiology and Biostatistics, and Psychiatry, University of California at San Francisco, San Francisco, California, USA **Urs Berger**, PhD, Associate Professor, Department of Applied Environmental Science, Stockholm University, Stockholm, Sweden **Åke Bergman**, PhD, Executive Director and Professor, Swedish Toxicology Sciences Research Center, Södertälje, Sweden **Vladimir Beškoski**, PhD, Assistant Professor, Faculty of Chemistry, University of Belgrade, Belgrade, Serbia **Emmanuelle Bichon**, Scientific and Technical Support Manager, Oniris, Nantes-Atlantic College of Veterinary Medicine, Food Science and Engineering, Nantes, France **Filip Bjurlid**, PhD Student, Man–Technology–Environment Research Centre, Örebro University, Örebro, Sweden **Tara Blank**, PhD, Consultant, Elixir Environmental, Ridgefield, Connecticut, USA **Daniel Borg**, PhD, Toxicology Consultant, Trossa AB, Stockholm, Sweden **Carl-Gustaf Bornehag**, PhD, Professor, Department of Health and Environment, Karlstad University, Karlstad, Sweden **Hindrik Bouwman**, PhD, Lecturer, Zoology Group, North-West University, Mahikeng, South Africa **Lindsay Bramwell**, MSc, Research Associate, Institute of Health and Society, Newcastle University, Newcastle upon Tyne, United Kingdom **Knut Breivik**, PhD, Senior Scientist and Professor, NILU–Norwegian Institute for Air Research, Kjeller, Norway **Katja Broeg**, PhD, Researcher, Baltic Sea Centre, Stockholm University, Stockholm, Sweden **Phil Brown**, PhD, University Distinguished Professor of Sociology and Health Sciences, and Director, Social Science Environmental Health Research Institute, Northeastern University, Boston, Massachusetts, USA **Thomas Bruton**, MS, PhD Student, Department of Civil and Environmental Engineering, University of California, Berkeley, Berkeley, California, USA **David Camann**, MS, Technical Advisor, Southwest Research Institute, San Antonio, Texas, USA **Louise Camenzuli**, PhD Student, Safety and Environmental Technology Group, Institute for Chemical and Bioengineering, ETH Zürich, Zürich, Switzerland **Argelia Castaño**, PhD, Head of Department, Area of Environmental Toxicology, Instituto de Salud Carlos III, Majadahonda, Spain **Carmela Centeno**, Industrial Development Officer, United Nations Industrial Development Organization, Vienna, Austria **Ibrahim Chahoud**, PhD, Professor, Department of Toxicology, Charité–Universitätsmedizin Berlin, Berlin, Germany **Kai Hsien Chi**, PhD, Associate Professor, Institute of Environmental and Occupational Health Sciences, National Yang-Ming University, Taipei, Taiwan **Eliza Chin**, MD, MPH, Executive Director, American Medical Women’s Association, Reston, Virginia, USA **Carsten Christophersen**, PhD, Adjunct Professor, Systems Biology, Technical University of Denmark, Kongens Lyngby, Denmark **Theo Colborn (1927–2014)**, PhD, President Emeritus, TEDX (The Endocrine Disruption Exchange), Paonia, Colorado, USA **Terrence J. Collins**, PhD, Teresa Heinz Professor of Green Chemistry, Department of Chemistry, Carnegie Mellon University, Pittsburgh, PA, USA; and Director, Institute for Green Science, Pittsburgh, Pennsylvania, USA **Johanna Congleton**, MSPH, PhD, Senior Scientist, Environmental Working Group, Washington, DC, USA **Adrian Covaci**, PhD, Professor, Toxicological Center, University of Antwerp, Antwerp, Belgium **Craig Criddle**, PhD, Professor, Department of Civil and Environmental Engineering, Stanford University, Stanford, California, USA **Oscar H. Fernández Cubero**, Technician, National Food Center, Majadahonda, Spain **Jordi Dachs**, PhD, Research Scientist, Institute of Environmental Assessment and Water Research, Spanish Council for Scientific Research, Barcelona, Spain **Cynthia de Wit**, PhD, Professor, Department of Applied Environmental Science, Stockholm University, Stockholm, Sweden **Barbara Demeneix**, PhD, DSc, Professor, Department RDDM, National Museum of Natural History, Paris, France **Pascal Diefenbacher**, PhD Student, Safety and Environmental Technology Group, Institute for Chemical and Bioengineering, ETH Zürich, Zürich, Switzerland **Michelle Douskey**, PhD, Chemistry Lecturer, Department of Chemistry, University of California, Berkeley, Berkeley, California, USA **Timothy Elgren**, PhD, Dean of Arts and Sciences, Oberlin College, Oberlin, Ohio, USA **David Epel**, PhD, Professor Emeritus, Hopkins Marine Station, Stanford University, Pacific Grove, California, USA **Ulrika Eriksson**, PhD Student, Man–Technology–Environment Research Centre, Örebro University, Örebro, Sweden **Alexi Ernstoff**, MS, PhD Student, Quantitative Sustainability Assessment, Technical University of Denmark, Kongens Lyngby, Denmark **Igor Eulaers**, PhD Student, Department of Biology, University of Antwerp, Antwerp, Belgium **Heesoo Eun**, PhD, Senior Researcher, Division of Organochemicals, National Institute for Agro-Environmental Sciences, Tsukuba, Japan **Peter Fantke**, PhD, Assistant Professor, Quantitative Sustainability Assessment Division, Department of Management Engineering, Technical University of Denmark, Kongens Lyngby, Denmark **Marko Filipovic**, FilLic, Department of Applied Environmental Science, Stockholm University, Stockholm, Sweden **Marie Frederiksen**, Researcher, Danish Building Research Institute, Aalborg University, Copenhagen, Denmark **Carey Friedman**, PhD, Postdoctoral Associate, Center for Global Change Science, Massachusetts Institute of Technology, Cambridge, Massachusetts, USA **Frederic Gallo**, PhD, Senior Expert, Regional Activity Center for Sustainable Consumption and Production, Barcelona, Spain **Joseph A. Gardella, Jr**, PhD, Distinguished Professor and John and Frances Larkin Professor of Chemistry, Department of Chemistry, University of Buffalo–The State University of New York, Buffalo, New York, USA **Stephen Gardner**, DVM, Veterinarian, Albany Animal Hospital, Richmond, California, USA **Caroline Gaus**, PhD, Professor, National Centre for Environmental Toxicology, The University of Queensland, Brisbane, Queensland, Australia **Wouter Gebbink**, PhD, Researcher, Department of Applied Environmental Science, Stockholm University, Stockholm, Sweden **David Gee**, PhD, Associate Fellow, Institute of Environment, Health, and Societies, Brunel University, Brunel, United Kingdom **Philip Germansdefer**, DHC Che, MS ChE, Director of International Sales and Marketing, Fluid Management Systems, Inc., Watertown, Massachusetts, USA **Bondi Nxuma Gevao**, PhD, Research Scientist, Kuwait Institute for Scientific Research, Safat, Kuwait **Melissa Gomis**, MS, PhD Student, Department of Environmental Science, Stockholm University, Stockholm, Sweden **Belen Gonzalez**, PhD Student, Institute of Environmental Assessment and Water Research, Spanish Council for Scientific Research, Barcelona, Spain **Peter Gringinger**, MSc, Principal, Cardno, Sassafras, Victoria, Australia **Adam Grochowalski**, PhD, Professor, Department of Analytical Chemistry, Krakow University of Technology, Krakow, Poland **Ramon Guardans**, Scientific Advisor, Ministry of Agriculture, Food and Environment, Madrid, Spain **Alexey Gusev**, PhD, Senior Scientist, European Monitoring and Evaluation Programme Meteorological Synthesizing Centre–East, Moscow, Russia **Arno Gutleb**, PhD, Project Leader, Department of Environment and Agro-Biotechnologies, Luxembourg Institute of Science and Technology, Belvaux, Luxembourg **Tenzing Gyalpo**, PhD Student, Safety and Environmental Technology Group, Institute for Chemical and Bioengineering, ETH Zürich, Zürich, Switzerland **Johannes Hädrich**, PhD, Head, Research Laboratory, European Union Reference Laboratory for Dioxins and PCBs in Feed and Food, Freiburg, Germany **Helen Håkansson**, PhD, Professor of Toxicology and Chemicals Health Risk Assessment, Institute of Environmental Medicine, Karolinska Institutet, Stockholm, Sweden **Tomas Hansson**, PhD, Researcher, Department of Applied Environmental Science, Stockholm University, Stockholm, Sweden **Mikael Harju**, PhD, Senior Scientist, NILU–Norwegian Institute for Air Research, Tromsø, Norway **Stuart Harrad**, PhD, Professor of Environmental Chemistry, School of Geography, Earth and Environmental Sciences, University of Birmingham, Edgbaston, United Kingdom **Bernhard Hennig**, PhD, Professor of Nutrition and Toxicology, and Director, University of Kentucky Superfund Research Center, Lexington, Kentucky, USA **Eunha Hoh**, PhD, Associate Professor, Department of Public Health, San Diego State University, San Diego, California, USA **Sandra Huber**, PhD, Senior Researcher, Environmental Chemistry, NILU–Norwegian Institute for Air Research, Tromsø, Norway **François Idczak**, Direction de la Surveillance de l’Environnement, Institue Scientifique de Service Public (ISSeP), Liege, Belgium **Alastair Iles**, SJD, Associate Professor, Department of Environmental Science, Policy, and Management, University of California, Berkeley, Berkeley, California, USA **Ellen Ingre-Khans**, MSc, PhD Student, Department of Applied Environmental Science, Stockholm University, Stockholm, Sweden **Alin Constantin Ionas**, PhD Candidate, Toxicological Center, University of Antwerp, Antwerp, Belgium **Griet Jacobs**, Researcher, Flemish Institute of Technological Research, Mol, Belgium **Annika Jahnke**, PhD, Researcher, Department of Cell Toxicology, Helmholtz Centre for Environmental Research, Leipzig, Germany **Veerle Jaspers**, PhD, Associate Professor, Department of Biology, Norwegian University of Science and Technology, Trondheim, Norway **Allan Astrup Jensen**, PhD, Research Director and CEO, Nipsect, Frederiksberg, Denmark **Javier Castro Jimenez**, PhD Research Scientist, Institute of Environmental Assessment and Water Research, Spanish Council for Scientific Research, Barcelona, Spain **Ingrid Ericson Jogsten**, PhD, Research Scientist, School of Science and Technology, Örebro University, Örebro, Sweden **Jon E. Johansen**, Dr techn, Director, Chiron AS, Trondheim, Norway **Niklas Johansson**, Senior Consultant, Melica Biologkonsult, Upplands Väsby, Sweden **Paula Johnson**, PhD, MPH, Research Scientist, California Department of Public Health, Richmond, California, USA **Jill Johnston**, PhD, Postdoctoral Fellow, Department of Epidemiology, University of North Carolina at Chapel Hill, Chapel Hill, North Carolina, USA **Olga-Ioanna Kalantzi**, PhD, Assistant Professor, University of the Aegean, Mytilene, Greece **Anna Kärrman**, PhD, Associate Professor, Man–Technology–Environment Research Centre, Örebro University, Örebro, Sweden **Naila Khalil**, MBBS, MPH, PhD, Assistant Professor, Boonshoft School of Medicine, Wright State University, Kettering, Ohio, USA **Maja Kirkegaard**, PhD, Cand Scient, Research Advisory, Head of Chemicals Group, Worldwatch Institute Europe, Copenhagen, Denmark **Jana Klanova**, PhD, Professor, Research Center for Toxic Compounds in the Environment, Faculty of Science, Masaryk University, Brno, Czech Republic **Susan Klosterhaus**, PhD, Vice President, Science and Certification, Cradle to Cradle Products Innovation Institute, San Francisco, California, USA **Candice Kollar**, LEED AP, Design Strategist, Kollar Design | EcoCreative, San Francisco, California, USA **Janna G. Koppe**, PhD, Professor Emeritus of Neonatology, Emma Children’s Hospital/Academic Medical Center, University of Amsterdam, Loenersloot, the Netherlands **Ingjerd Sunde Krogseth**, PhD, Postdoctoral Fellow, NILU–Norwegian Institute for Air Research, Tromsø, Norway **Petr Kukucka**, PhD, Junior Researcher, Research Center for Toxic Compounds in the Environment, Faculty of Science, Masaryk University, Brno, Czech Republic **Perihan Binnur Kurt Karakus**, PhD, Associate Professor, Department of Environmental Engineering, Bursa Technical University, Bursa, Turkey **Henrik Kylin**, PhD, Professor, Department of Thematic Studies—Environmental Change, Linköping University, Linköping, Sweden **Remi Laane**, PhD, Professor, Department of Environmental Chemistry, University of Amsterdam, Deltares, Voorburg, the Netherlands **Jon Sanz Landaluze**, PhD, Assistant Professor, Department of Analytical Chemistry, Universidad Complutense de Madrid, Madrid, Spain **Le Thi Hai Le**, PhD, Department Deputy Director, Ministry of Natural Resources and Environment, Ha Noi, Vietnam **Jong-Hyeon Lee**, PhD, Director, NeoEnBiz, Gyeonggi-do, South Korea **Marike Martina Leijs**, PhD, Professor, Department of Dermatology, University Hospital RWTH Aachen, Aachen, Germany **Xiaodong Li**, PhD, Professor, Faculty of Engineering, Zhejiang University, Hangzhou, China **Yifan Li**, PhD, Professor, International Joint Research Center for Persistent Toxic Substances, Harbin Institute of Technology, Harbin, China **Danuta Ligocka**, PhD, Senior Researcher, Department of Toxicology and Carcinogenesis, Nofer Institute of Occupational Medicine, Łódź, Poland **Monica Lind**, PhD, Scientist, Occupational and Environmental Medicine, Uppsala University, Uppsala, Sweden **Lee Lippincott**, PhD, Assistant Professor of Chemistry, Allied Health Sciences, Mercer County Community College, West Windsor, New Jersey, USA **Mariann Lloyd-Smith**, PhD, Senior Advisor, National Toxics Network, East Ballina, New South Wales, Australia **Karin Löfstrand**, PhD, Postdoctoral Fellow, Department of Applied Environmental Science, Stockholm University, Stockholm, Sweden **Rainer Lohmann**, PhD, Associate Professor, Graduate School of Oceanography, University of Rhode Island, Kingston, Rhode Island, USA **Donald Lucas**, PhD, Research Scientist, Lawrence Berkeley National Laboratory, Berkeley, California, USA **José Vinicio Macias**, PhD, Researcher, Autonomous University of Baja California, Baja California, Mexico **Karl Mair**, Magister, Senior Environmental Chemist, Eco Research, Bolzano, Italy **Govindan Malarvannan**, PhD, Research Scientist, Faculty of Pharmaceutical, Biomedical and Veterinary Sciences, University of Antwerp, Antwerp, Belgium **Svetlana Malysheva**, PhD, Research Scientist, Scientific Institute of Public Health, Ghent University, Brussels, Belgium **Jonathan Martin**, PhD, Professor, Division of Analytical and Environmental Toxicology, University of Alberta, Edmonton, Alberta, Canada **Lisa Mattioli**, MSc, Scientist, Department of Chemistry, Carleton University Ottawa, Ontario, Canada **Michael McLachlan**, PhD, Professor, Department of Applied Environmental Science, Stockholm University, Stockholm, Sweden **Lisa Melymuk**, PhD, Junior Researcher, Research Center for Toxic Compounds in the Environment, Faculty of Science, Masaryk University, Brno, Czech Republic **Annelle Mendez**, PhD Student, Safety and Environmental Technology Group, Institute for Chemical and Bioengineering, ETH Zürich, Zürich, Switzerland **Tom Muir**, MS, Consultant (retired), Environment Canada, Burlington, Ontario, Canada **Marie Danielle Mulder**, PhD Student, Research Center for Toxic Compounds in the Environment, Faculty of Science, Masaryk University, Brno, Czech Republic **Jochen Müller**, PhD, Professor, National Research Centre for Environmental Toxicology, The University of Queensland, Brisbane, Queensland, Australia **Patricia Murphy**, ND, LAc, Naturopathic Physician, Portland, Oregon, USA **Takeshi Nakano**, PhD, Specially Appointed Professor, Graduate School of Engineering, Osaka University, Osaka, Japan **Amgalan Natsagdorj**, PhD, Associate Professor, Department of Chemistry, National University of Mongolia, Ulaanbaatar, Mongolia **Seth Newton**, PhD Student, Department of Applied Environmental Science, Stockholm University, Täby, Sweden **Carla Ng**, PhD, Senior Scientist, Safety and Environmental Technology Group, Institute for Chemical and Bioengineering, ETH Zürich, Zürich, Switzerland **Bo Normander**, PhD, Executive Director, Worldwatch Institute Europe, Copenhagen, Denmark **Kees Olie**, PhD, Retired, Institute for Biodiversity and Ecosystem Dynamics, Amsterdam, the Netherlands **Bindu Panikkar**, PhD, Research Associate, Arctic Institute of North America, Calgary, Alberta, Canada **Richard Peterson**, PhD, Professor, Department of Pharmaceutical Sciences, University of Wisconsin, Madison, Wisconsin, USA **Arianna Piersanti**, PhD, Lead Chemist, Food of Environmental Control Department, Istituto Zooprofilattico Sperimentale dell-Umbria e dell Marche, Perugia, Italy **Merle Plassmann**, PhD, Researcher, Department of Applied Environmental Science, Stockholm University, Stockholm, Sweden **Anuschka Polder**, PhD, Scientist, Department of Food Safety and Infection Biology, Norwegian University of Life Sciences, Oslo, Norway **Malte Posselt**, BSc, MS Student, German Federal Environment Agency, Berlin, Germany **Deborah O. Raphael,** Director, San Francisco Department of the Environment, San Francisco, California, USA **Shay Reicher**, PhD, Risk Assessment Director, Ministry of Health, Tel Aviv, Israel **Efstathios Reppas-Chrysovitsinos**, MEng, PhD Candidate, Department of Applied Environmental Science, Stockholm University, Stockholm, Sweden **Crystal Reul-Chen**, DEnv, Senior Environmental Scientist, California Environmental Protection Agency, Sacramento, California, USA **David Roberts**, PhD, Kenan Professor of Physics, Department of Physics, Brandeis University, Waltham, Massachusetts, USA **Mary Roberts**, PhD, Professor, Merkert Chemistry Center, Boston College, Chestnut Hill, Massachusetts, USA **Camilla Rodrigues**, PhD, Researcher, Environmental Sanitation Technology Company, San Paulo, Brazil **Ott Roots**, Dr sc nat ETH, Director of the Institute/Leading Research Scientist, Estonian Environmental Research Institute, Tallinn, Estonia **Maria Ros Rodriguez**, Laboratory Technician, Instituto de Química Orgánica General-Consejo Superior de Investigaciones Científicas, Madrid, Spain **Anna Rotander**, PhD, Postdoctoral Researcher, Man–Technology–Environment Research Centre, Örebro University, Örebro, Sweden; and National Research Centre for Environmental Toxicology, The University of Queensland, Brisbane, Queensland, Australia **Ruthann Rudel**, MS, Director of Research, Silent Spring Institute, Newton, Massachusetts, USA **Christina Rudén**, PhD, Professor, Department of Applied Environmental Science, Stockholm University, Stockholm, Sweden **Andreas Béguin Safron**, MSc, PhD Candidate, Department of Applied Environmental Science, Stockholm University, Stockholm, Sweden **Amina Salamova**, PhD, Research Scientist, School of Public and Environmental Affairs, Indiana University, Bloomington, Indiana, USA **Samira Salihovic**, PhD, Postdoctoral Fellow, Department of Medical Sciences, Uppsala University, Uppsala, Sweden **Johanna Sandahl**, MS, President, Swedish Society for Nature Conservation, Stockholm, Sweden **Erik Sandell**, Consulting Specialist, Nab Labs Oy, Espoo, Finland **Andreas Schaeffer**, PhD, Institute Director, Institute for Environmental Research, RWTH Aachen University, Aachen, Germany **Julia Schaletzky**, PhD, Senior Group Leader, Cytokinetics, South San Francisco, California, USA **Arnold Schecter**, PhD, Professor, School of Public Health, University of Texas–Dallas Campus, Dallas, Texas, USA **Ted Schettler**, MD, MPH, Science Director, Science and Environmental Health Network, Ames, Iowa, USA **Margret Schlumpf**, Dr sc nat ETH, Co-Director, Group for Reproductive, Endocrine and Environmental Toxicology, University of Zürich, Zürich, Switzerland **Peter Schmid**, PhD, Senior Scientist, Department of Organic Chemistry, Swiss Federal Institute for Material Research and Testing, Dübendorf, Switzerland **Lara Schultes**, MSc, PhD Student, Department of Applied Environmental Science, Stockholm University, Stockholm, Sweden **Susan Shaw**, PhD, Professor, School of Public Health, University at Albany–State University of New York, Albany, New York, USA; and Director, Marine Environmental Research Institute, Blue Hill, Maine, USA **Omotayo Sindiku**, Research Assistant, Basel Convention Coordinating Center, Ibadan, Nigeria **Line Småstuen Haug**, PhD, Senior Scientist, Department of Exposure and Risk Assessment, Norwegian Institute of Public Health, Oslo, Norway **Anna Sobek**, PhD, Researcher, Department of Applied Environmental Science, Stockholm University, Stockholm, Sweden **Ana Sousa**, PhD, Postdoctoral Researcher, Health Sciences Research Centre, University of Beira Interior, Covilhã, Portugal **Martin Sperl**, Technician, Austria Metall AG, Ranshofen, Austria **Thomas Steiner**, PhD, CEO, MonitoringSystems GmbH, Pressbaum, Austria **Christine Steinlin**, PhD Student, Safety and Environmental Technology Group, Institute for Chemical and Bioengineering, ETH Zürich, Zürich, Switzerland **Alex Stone**, ScD, Senior Chemist, Hazardous Waste and Toxics Reduction Program, Washington State Department of Ecology, Lacey, Washington, USA **William Stubbings**, PhD Student, University of Birmingham, Edgbaston, United Kingdom **Roxana Sühring**, PhD Student, Helmholtz-Zentrum Geesthacht, Lüneburg, Germany **Kimmo Suominen**, PhD, Senior Researcher, Finish Food Safety Authority, Risk Assessment Research Unit, Helsinki, Finland **Rebecca Sutton**, PhD, Senior Scientist, San Francisco Estuary Institute, Richmond, California, USA **Joel Svedlund**, BSc, Sustainability Manager, Klättermusen AB, Åre, Sweden **David Szabo**, PhD, Senior Scientist, Research and Development, Reynolds American, Winston-Salem, North Carolina, USA **Öner Tatli**, Lab Manager, A&G P¸r Analysis Laboratory, Izmir, Turkey **Neeta Thacker**, MSc, PhD, Former Chief Scientist and Quality Manager, Analytical Instruments Division, National Environmental Engineering Research Institute, Nagpur, India **Dien Nguyen Thanh**, PhD Student, Environment Preservation Research Center, Kyoto University, Kyoto, Japan **Joao Paulo Machado Torres**, PhD, Associate Professor, Instituto de Biofisica Carlos Chagas Filho, Rio de Janeiro Federal University, Rio de Janeiro, Brazil **Matthew Trass**, PhD, Research Scientist, Phenomenex, Torrance, California, USA **Theodora Tsongas**, PhD, MS, Environmental Health Scientist and Consultant, Portland, Oregon, USA **Mary Turyk**, PhD, Associate Professor, Department of Epidemiology and Biostatistics, University of Illinois at Chicago, Chicago, Illinois, USA **Anthony C. Tweedale**, MS, Consultant, Rebutting Industry Science with Knowledge Consultancy, Eastpointe, Michigan, USA **Marta Venier**, PhD, Scientist, School of Public and Environmental Affairs, Indiana University, Bloomington, Indiana, USA **Robin Vestergren**, PhD, Postdoctoral Researcher, Environmental Chemistry, NILU–Norwegian Institute for Air Research, Tromsø, Norway **Stefan Voorspoels**, PhD, Research Manager, Flemish Institute of Technological Research, Mol, Belgium **Shu-Li Wang**, PhD, Investigator and Professor, Department of Environmental Health and Occupational Medicine, National Health Research Institute, Chunan, Miaoli, Taiwan **Glenys Webster**, PhD, Postdoctoral Fellow, Developmental Neurosciences and Child Health, Child and Family Research Institute, and Faculty of Health Sciences, Simon Fraser University, Vancouver, British Columbia, Canada **Larry Weiss**, MD, Chief Marketing Officer, AOBiome, LLC, San Francisco, California, USA **Philip White**, Organics Analyst, Marine Institute, Galway, Ireland **Karin Wiberg**, PhD, Professor, Department of Aquatic Sciences and Assessment, Swedish University of Agricultural Sciences, Uppsala, Sweden **Gayle Windham**, PhD, Research Scientist, Division of Environmental and Occupational Health Control, California Department of Public Health, Richmond, California, USA **Hendrik Wolschke**, PhD Student, Helmholtz Zentrum Geesthacht-Centre for Materials and Coastal Research, Geesthacht, Germany **Bo Yuan**, PhD, Postdoctoral Fellow, Department of Applied Environmental Science, Stockholm University, Stockholm, Sweden **Elena Zaffonato**, Organics Analyst, Chelab Sri, Resana Treviso, Italy **Lingyan Zhu**, PhD, Professor, College of Environmental Science and Engineering, Nankai University, Tianjin, China **Robert Zoeller**, PhD, Professor, Department of Biology, University of Massachusetts Amherst, Amherst, Massachusetts, USA
